# Whole-genome-based phylogeny of African swine fever virus

**DOI:** 10.14202/vetworld.2020.2118-2125

**Published:** 2020-10-10

**Authors:** Levon Aslanyan, Hranush Avagyan, Zaven Karalyan

**Affiliations:** 1 Department of Mathematics, Institute for Informatics and Automation Problems of NAS RA, Yerevan, Armenia; 2 Laboratory of Cell Biology and Virology, Institute of Molecular Biology of NAS RA, Yerevan, Armenia; 3 Experimental Laboratory, Yerevan State Medical University, Yerevan, Armenia; 4 Department of Medical Biology, Yerevan State Medical University, Yerevan, Armenia

**Keywords:** African swine fever virus, baculovirus, phylogenetic tree

## Abstract

**Aim:**

A genome-scale phylogenetic analysis was used to infer the evolutionary dynamics of *Asfarviridae* – African swine fever virus (ASFV) – and better define its genetic diversity.

**Materials and Methods:**

All complete ASFV genomes from NCBI’s resource as of March 2020 were used. The phylogenetic analysis used maximum likelihood and neighbor-joining methods. The evolutionary models detection was done with the help of the package of programs MEGA-X. Algorithms were used to build phylogenetic trees for type B DNA polymerases of ASFV (n=34) and HcDNAV (n=2), as an external group.

**Results:**

An expedient categorization of the Asfarviridae family uses five clades. Genotype 1 (except for LIV 5/40 virus isolate) as well genotype 7 are assigned to the alpha clade; genotype 2 to the beta clade; genotypes 8, 9, and 10 to the gamma clade; genotype 5 to the delta clade; and genotypes 3, 4, and 20, as well as genotype 22 and the LIV 5/40 isolate to the epsilon clade. Branch lengths on the phylogenetic tree are proportional to genetic distance along the branch. Branches at the phylogenetic tree of Asfarviridae are much shorter than branches for Baculoviridae. Shorter branches in ASFVs population suggest that Asfarviridae evolved relatively recently and remain more closely related.

**Conclusion:**

We suggest applying more robust standards using whole genomes to ensure the correct classification of ASFV and maintain phylogeny as a useful tool.

## Introduction

Viral genomes usually evolve rapidly, and ­accumulated changes, through either mutation or recombination with other strains or species, are first fixed in the genome of successful virus isolates that give rise to genetic lineages. The relationship between biological lineages related by common descent is called “phylogeny.” When the history of evolution is coded from ancestry, we derive a tree with the root as an ancestor. In contrast, we are often given some population of viruses to solve the reverse problem to describing the steps of the process [1]. In such case, we adopt some parametric characterization of mutations and recombination from which phylogeny can be inferred. A complex mathematical framework has been developed for phylogenic inferences. The framework examines interspecies differences followed by phylogenic tree deduction and comparison. A reconstructed tree approximates the “true” phylogeny that generally remains unknown. The phylogenetic analysis is used in applied and basic virological research, including epidemiology, diagnostics, forensic studies, phylogeography, evolutionary studies, and virus taxonomy. The analyses provide an evolutionary perspective on the variation in any trait that can be measured for a group of viruses [1].

The origin and evolution of Asfarviridae are of special interest as common agricultural pathogens. The diversity of these viruses – 24 genotypes – imposes difficulties in collective evaluation of phylogenetic relationships. Modern phylogenic diversity of African swine fever virus (ASFVs) is based on the sequence of the p72 (B646L) gene that allows identification of the variation level [2,3].

The issue of the modern evolution of Asfarviridae is of great interest, and a general understanding is recognized. The major hindrance for phylogenetic exploration is the isolation of this group of viruses. Viruses have no universal genes analogous to ribosomal RNA genes of all cellular organisms, reconstruct the phylogenetic tree, as well as evolution roots for distinct virus groups, and lack a firm baseline.

We conducted a genome-scale phylogenetic analysis to infer the evolution of ASFV and to define evolutionary dynamics and genetic diversity better. The current analysis also includes another family of closely related DNA viruses – the Baculoviridae and Asfarviridae. Sequences were analyzed using a set of publicly available whole genomes previously ­deposited in GenBank. Whole-genome analysis intends to provide more robust and comprehensive information on ASF virus evolution and assessment of evolutionary history and relationships among distinct isolates.

## Materials and Methods

### Ethical approval

The ethics approval proposal was not required because the data were collected from the GenBank sequence database in NCBI. Research was conducted ethically in accordance with the World Medical Association Declaration of Helsinki.

### Study period and location

The study started in early October 2019 and finished in late March 2020. The study was conducted in Laboratory of Cell Biology and Virology, Institute of Molecular Biology of NAS RA.

### Sequence acquisition and metadata curation

We downloaded all complete ASFV genomes from NCBI’s Resource as of March 2020 (n=60). All supporting information, including host, country, and date of isolation, and genotype was recorded. The approximate length of the genomes of this family is in order of 200,000.

### Phylogenetic analysis

Several sets of complete genomes were separately aligned using Clustal Omega (https://www.ebi.ac.uk/Tools/msa/) and similar algorithms, such as Muscle and Mafft (general purpose multiple sequence alignment programs for DNA sequences), to build phylogenetic trees for type B DNA polymerases of ASFV (n=34) and HcDNAV (n=2), as an external group.

Evolutionary relationships were assessed using the MEGA-X program’s package [4]. Parameters were preserved by default. Phylogenetic trees were constructed with the maximal likelihood approach available in the list of MEGA-X models.

The best known similar/dissimilar measure between sequences is the longest common subsequence (LCS), even though the actual LCS is hardly computable. LCS algorithms are regarded as NP hard problems in combinatorial interpretation, which are studied for finding successful polynomial algorithmic solutions [5,6]. The known approximations to LCS are performed through the *k-mer*, FFT, and other combinatorial means. The pairwise distances for *m* sequences can be computed by different runs, or it might be approximated by an integrative multiple alignment computation. The Sequence Demarcation Tool http://web.cbio.uct.ac.za/~brejnev/SDTv1.2 recommends the pairwise computations that are time-consuming, though tractable on supercomputers. The problem is in length, *l*, of the genome. Complexities of computation and testing for recombination increase exponentially with the number of sequences *m* and increase linearly with the lengths sequences examined. However, even the linearity of *l* complicates computations often making it practically impossible. Multiple sequence alignment is also time-consuming, yet, it provides an acceptable approximation for 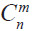 pairwise distances in one run. Our strategy is to apply this approximation with testing in narrow sites. The set of sequences was narrowed by deleting evidently classified sequences and keeping the neighborhood of the suspicious join, when encountering any phylogenetic join that was subject to the test. We then computed the actual pairwise distances, scaling and checking the distance differences in MSM and LCS.

## Results

### Whole-genome alignment of ASFV isolates

The complete genomes of all 60 ASF viruses (available in GenBank) were successfully sequenced in this study (Table-1, data with identical sequences were removed) [7-22]. In addition, all available (by March 2020) ASFV genomes were downloaded and included in all analyses (see alignment in the supplementary material).

**Table 1:** African swine fever virus whole-genome sequences available from public databases (status March 31, 2020)*.

Isolate	Genotype	References
ASFV isolate 26544/OG10 from Italy complete genome	I	[8]
ASFV isolate 47/Ss/2008 complete genome	I	[9]
ASFV Benin 97/1 pathogenic isolate complete genome	I	[7,10]
ASFV E75 complete genome strain E75	I	[7]
ASFV strain L60 complete genome	I	[11]
ASFV strain BA71V complete genome	I	[12]
ASFV strain NHV complete genome	I	[11]
ASFV OURT 88/3 (avirulent field isolate) complete genome	I	[10]
ASFV isolate Mkuzi 1979 complete genome	VII	Kutish G.F. and Rock D.L. N/A: Data not available;
ASFV strain Liv13/33 complete genome	I	Chastagner A., Le Potier M.-F. and Pereira de Oliveira, R. N/A: Data not available;
ASFV strain Belgium/Etalle/wb/2018 complete genome	II	[13]
ASFV isolate Pig/HLJ/2018 complete genome	II	[14]
ASFV isolate DB/LN/2018 complete genome	II	[14]
ASFV strain ASFV HU 2018 complete genome	II	[15]
ASFV isolate ASFV-wbBS01 complete genome	II	Zhaowen R., Huancheng G., and Changchun T. Genome comparison of ASFV ASFV-wbBS01 strain and different from ASFV-SY18 from domestic pig. unpublished
ASFV isolate ASFV Moldova 2017/1 genome assembly complete genome	II	[16]
ASFV isolate ASFV/Kyiv/2016/131 complete genome	II	[17]
ASFV isolate ASFV/LT14/1490 complete genome	II	[18]
ASFV strain Georgia 2008/1 complete genome	II	[19]
ASFV Georgia 2007/1 complete genome	II	[20]
ASFV isolate Malawi Lil-20/1 (1983) complete genome	VIII	Kutish G.F. and Rock D.L. N/A: Data not available
ASFV strain Ken05/Tk1 complete genome	IX	[21]
ASFV isolate Kenya 1950 complete genome	X	Kutish G.F. and Rock D.L. N/A: Data not available
ASFV strain Ken06.Bus complete genome	X	[21]
ASFV strain R7 complete genome	IX	[16]
ASFV strain R8 complete genome	IX	[22]
ASFV strain R35 complete genome	IX	[22]
ASFV strain R25 complete genome	IX	[22]
ASFV strain N10 complete genome	IX	[22]
ASFV isolate Tengani 62 complete genome	V	Kutish, G.F. and Rock, D.L. N/A: Data not available
ASFV isolate Pretorisuskop/96/4 complete genome	XX	Kutish, G.F. and Rock, D.L. N/A: Data not available
ASFV isolate Warthog complete genome	IV	Kutish, G.F. and Rock, D.L. N/A: Data not available
ASFV isolate Warmbaths complete genome	III	Kutish, G.F. and Rock, D.L. N/A: Data not available
ASFV isolate SPEC 57 complete genome	III	[7]
ASFV isolate RSA 2/2008 complete genome	XXII	[7]
ASFV isolate LIV 5/40 complete genome	I	[7]

N/A=Data not available.

* Identical sequences were not included in table

Some perturbation is applied to check the stability of such disposition through elimination of specific sequences over the set of considered sequences. However as the general clusterization is inside the phylogeny, such perturbation may have an insignificant impact. At the same time, it can provide a valid tool to test sequence similarities and variations in these similarities.

### Phylogenetic analyses of ASFV isolates

**Figure-1:**
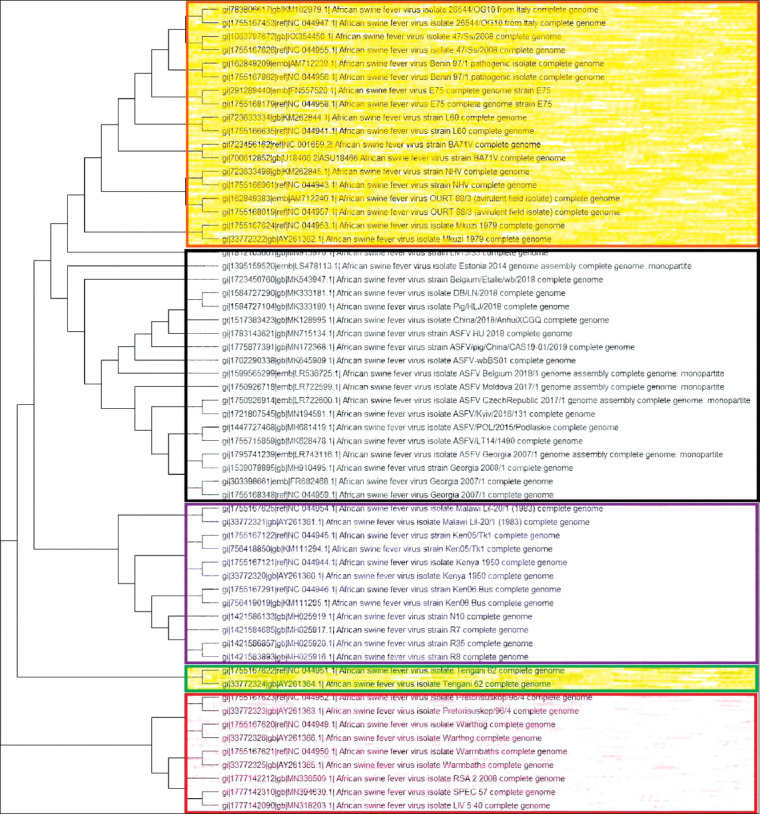
Phylogenic tree of Asfaviridae. Phylogenetic tree showing all available ASFV complete genome sequences (n=60). Orange color – alpha clade; gray color – beta clade; violet color – gamma clade; green color – delta clade; red color – epsilon clade.

Evolutionary history was inferred using the neighbor-joining method [23]. The optimal tree with the sum of branch length=2.12231299–is shown in Figure-1. The tree is drawn to scale, with branch lengths (next to the branches) in the same units as those of the evolutionary distances used to infer the tree. The evolutionary distances were computed using the p-distance method [24] and are in the units of the number of base differences per site. This analysis involved 20 nucleotide sequences. All ambiguous positions were removed for each sequence pair (pairwise deletion option). A total of 281,332 positions were included in the final dataset. Evolutionary analyses were conducted in MEGA-X [4].

The percentage of replicate trees in which the associated taxa clustered together in the bootstrap test (500 replicates) is shown next to the branches [25].

The phylogenetic tree analysis derived from ASFV complete genomes (Table-1) separated all sequenced viruses into five clades (Figure-1). The first clade (alpha) consists of all viruses with genotype 1, except Liv 5/40. This clade also includes viruses from genotype 7 – the Mkuzi 1979 isolate. The second clade (beta) presents all viruses with genotype 2. The third clade (gamma) consists of all viruses of genotypes 8, 9, and 10. The fourth clade (delta) consists of only genotype 5. The fifth clade (epsilon) consists of viruses from genotypes 3, 4, 20, and 22 and virus Liv 5/40. This isolated is regarded as genotype 1 by p72 classification [7].

**Figure-2:**
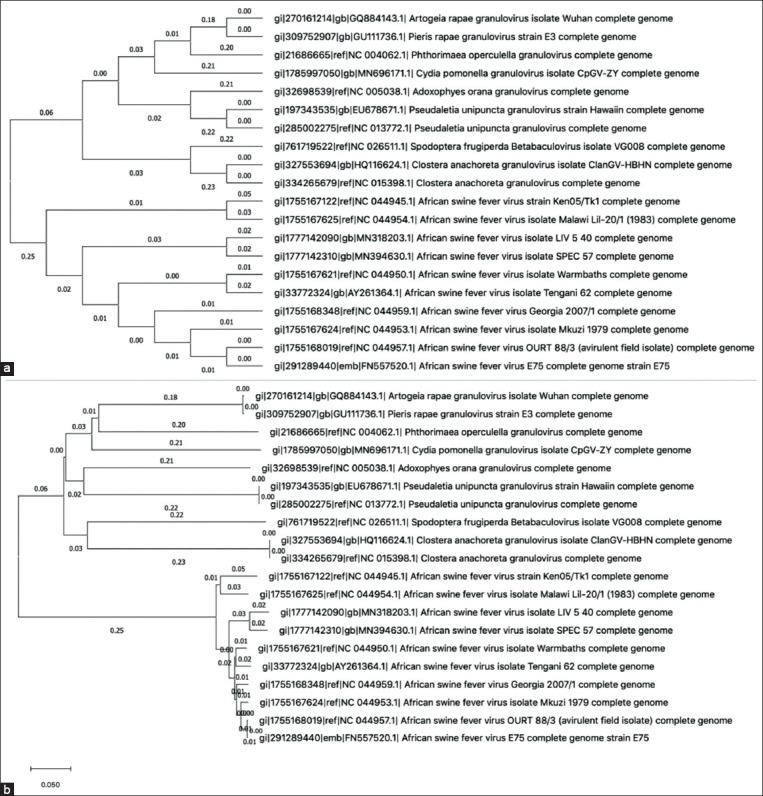
Phylogenetic analysis of Asfarviridae compared with Baculoviridae as external group. (a) Phylogenetic tree showing topology between Baculoviridae and Asfarviridae. (b) Baculoviridae and Asfarviridae phylogenetic tree with real branch sizes.

Asfarviridae*:* We compared the above tree with the closest related (by whole-genome analysis) virus clade – Baculoviridae [26] to further analyze the phylogeny of the Asfarviridae. These data are presented in Figures-2a and b. Baculoviridae and Asfarviridae are clustered into two different groups. The branches of the phylogenetic tree of Asfarviridae are much shorter than those in Baculoviridae (Figure-2b).

### Comparison between phylogeny based on the whole-genome analysis and phylogeny based on p72

Two main differences exist between the phylogenesis based on analysis of the complete genome and conventional phylogenesis based on the p72 (B646L) gene.

First, the placement of genotype 7 is inside the clade of genotype 1 using the p72 gene. This phenomenon was described previously [27]. Second, Liv 5/40, formerly referred to as genotype 1 by p72 [7], is in clade epsilon along with genotypes 3, 4, and 20.

**Figure-3:**
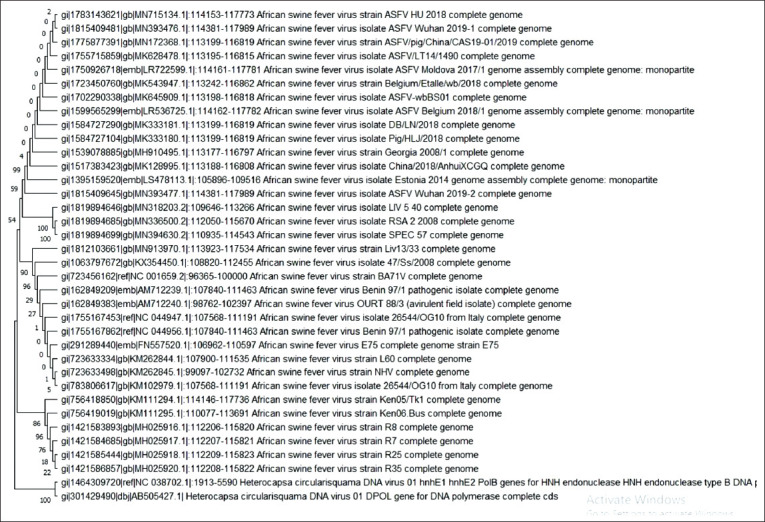
Comparative phylogenetic analysis between the DNA pol genes of African swine fever virus (n=34) and HcDNAV (n=2). All nodes were supported by bootstrap values (10,000 replicates).

We also processed phylogeny of DNA polymerase ASFV genes and included type B DNA polymerase (PolB) gene of HcDNAV, a virus that is closely related to ASFV [28], to study Liv 5/40 phenomenon further. This measurement showed that the Liv 5/40 isolate clusters with SPEC 57 and RSA-2 isolates (i.e., with genotypes 3 and 22) (Figure-3).

### Analysis of spatial genetic variation of ASFV populations of the African continent before 2007

**Figure-4:**
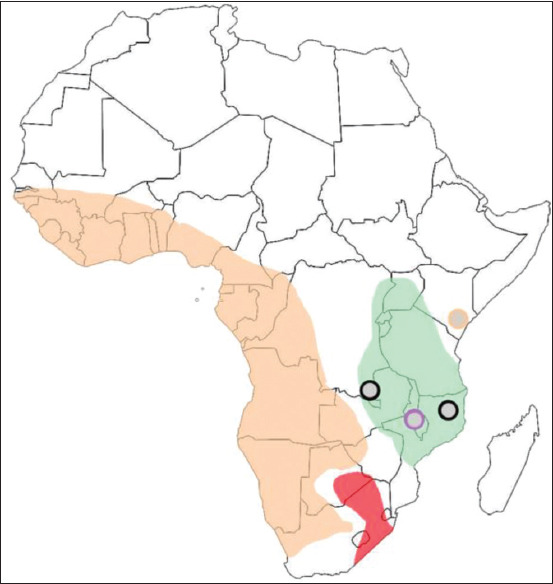
Geographical distribution of the Asfarviridae clades in Africa. Orange color – alpha clade; gray color – beta clade; violet color – gamma clade; green color – delta clade; red color – epsilon clade.

We used data [29,30] on the spatial distribution of various genotypes of ASFV (based on the *B646L* gene) in the African continent before the 2007 ­epidemic to determine the geographical boundaries of ASFV clades. Clade alpha is found in the Western parts of Africa. Clade beta was only distributed in East Africa (until 2007). Clade gamma is also identified in East Africa. Clade delta was found in Central and East Africa, and clade epsilon was found in the south and partially in the eastern areas of Africa (Figure-4).

## Discussion

The phylogenetic tree based on ASFV complete genomes is not precisely clear. Current genetic typing of ASFV isolates is generally based on nucleotide sequencing of the p72 capsid protein gene [31,32]. This classification enables the rapid identification of the virus. However, the correlation between ASFV genotypes (based on p72 classification) and viral cross-protection does not always match [33]. The Ugandan 1965 isolate is placed in VP72 genotype X, but it is placed in the genotype I clade [34].

The phylogenetic tree based on all available nucleotide sequences of the ASFV complete genomes revealed the genetic relationship between different virus genotypes obtained from various geographic areas and hosts. These relationships indicate that:

1. It is most expedient to divide the entire Asfarviridae family into five clades. Genotype 1 is assigned to the alpha clade (without the LIV 5/40 virus); this clade also includes genotype 7. This phenomenon was described previously [27]. Genotype 2 is assigned to the beta clade; genotypes 8, 9, and 10 to the gamma clade, genotype 5 to the delta clade; and genotypes 3, 4, and 20 to the epsilon clade, along with genotype 22 and the LIV 5/40 isolate

An alternative comparative phylogenetic analysis of the ASFV p72 gene suggested four clades [35]. Clade A was found in Central and East Africa and included genotypes 9 and 10. Clade B was distributed in East Africa and included genotypes 8 and 11-16. Clade C was identified in both East and South Africa and included genotypes 1, 2, 17, and 18. Genotypes included in clade D (3-7 and 19-22) were found in the Southern part of Africa, in Mozambique and Malawi.

Another alternative phylogeny was suggested by Wang *et al*. [36]. Our data largely coincide with this phylogeny. Genotype 2 in the latter study is localized into a separate group, and genotypes 8, 9, and 10 are clustered together. Some differences are explained by genome-wide analysis in our work, and our decision is to divide the Asfarviridae family into five clades, not in three.

1. Clade D described by Muangkram *et al*. [35], almost corresponds to our epsilon group. It includes viral populations in South and East Africa and is represented by genotypes 3, 4, 20, and 22.
2. ASF virus LIV 5/40, classified based on p72 (B646L) to genotype 1 [7], clusters together with genotypes 3 and 22 in the whole-genome analysis and, is quite close to genotypes 4 and 20
3. Various classifications indicating a lack of congruence between whole genomic analysis and p72-based single gene analysis were first postulated by de Villiers *et al*. [27]. Our data confirm this inference
4. Lengths of phylogenetic tree branches reflect genetic distances. Therefore, shorter branches in Asfarviridae versus Baculoviridae indicate that these viruses had fewer changes and are considered more closely related.

Shorter branches are found in populations with reduced genetic diversity, and this rule is applicable at both species and molecular levels [37,38]. Hence, we can conclude that shorter branches of ASFV clade represent a shorter period of evolution, or, perhaps, Baculoviridae evolve faster than Asfarviridae. However, modern literature sources [39] indicate that a longer evolution period of evolution is more likely. Analysis using whole genomes can allow more accurate evaluation of virus evolution rate.

Further, whole-genome sequencing of isolates sampled from various dsDNA viruses may provide better understanding of evolutionary and selection processes and more accurate estimates of divergence times and origins of distinct DNA viruses and separate viral genes. Continued surveillance and sequencing of different ASFV isolates is important for monitoring this virus family.

## Conclusion

We suggest applying more robust standards based on whole-genomes analysis to ensure the correct classification of ASFV to maintain phylogenetic analysis as a useful tool. Our data suggest an expedient classification of the entire Asfarviridae family into five clades. Shorter branches in ASFVs phylogenetic tree versus Baculoviridae suggest that Asfarviridae arose more recently.

## Authors’ Contributions

LA and ZK were responsible for the conception and design. HA coordinated the analysis. LA and ZK were responsible for the conclusive and final remarks. ZK did the final approval along with researchers. All authors read and approved the final manuscript.
